# Electrophysiological Guidance of Epidural Electrode Array Implantation over the Human Lumbosacral Spinal Cord to Enable Motor Function after Chronic Paralysis

**DOI:** 10.1089/neu.2018.5921

**Published:** 2019-04-12

**Authors:** Jonathan S. Calvert, Peter J. Grahn, Jeffrey A. Strommen, Igor A. Lavrov, Lisa A. Beck, Megan L. Gill, Margaux B. Linde, Desmond A. Brown, Meegan G. Van Straaten, Daniel D. Veith, Cesar Lopez, Dimitry G. Sayenko, Yury P. Gerasimenko, V. Reggie Edgerton, Kristin D. Zhao, Kendall H. Lee

**Affiliations:** ^1^ Mayo Clinic Graduate School of Biomedical Sciences, Mayo Clinic, Rochester, Minnesota.; ^2^ Department of Neurologic Surgery, Mayo Clinic, Rochester, Minnesota.; ^3^ Department of Physical Medicine and Rehabilitation, Rehabilitation Medicine Research Center, Mayo Clinic, Rochester, Minnesota.; ^4^ Department of Integrative Biology and Physiology University of California Los Angeles, Los Angeles, California.; ^5^ Pavlov Institute of Physiology, St. Petersburg, Russia.; ^6^ Department of Neurobiology, University of California Los Angeles, Los Angeles, California.; ^7^ Department of Neurosurgery, University of California Los Angeles, Los Angeles, California.; ^8^ Brain Research Institute, University of California Los Angeles, Los Angeles, California.; ^9^ Institut Guttmann, Hospital de Neurorehabilitació, Institut Universitari adscrit a la Universitat Autònoma de Barcelona, Barcelona, Badalona, Spain.; ^10^ Centre for Neuroscience and Regenerative Medicine, Faculty of Science, University of Technology Sydney, Ultimo, New South Wales, Australia.; ^11^ Department of Physiology and Biomedical Engineering, Mayo Clinic, Rochester, Minnesota.; ^12^ Center for Neuroregeneration, Department of Neurosurgery, Houston Methodist Research Institute, Houston, Texas.

**Keywords:** electrically evoked spinal motor potentials, epidural electrical stimulation, spinal cord injury, neuromodulation, spinal cord intraoperative electrophysiology

## Abstract

Epidural electrical stimulation (EES) of the spinal cord has been shown to restore function after spinal cord injury (SCI). Characterization of EES-evoked motor responses has provided a basic understanding of spinal sensorimotor network activity related to EES-enabled motor activity of the lower extremities. However, the use of EES-evoked motor responses to guide EES system implantation over the spinal cord and their relation to post-operative EES-enabled function in humans with chronic paralysis attributed to SCI has yet to be described. Herein, we describe the surgical and intraoperative electrophysiological approach used, followed by initial EES-enabled results observed in 2 human subjects with motor complete paralysis who were enrolled in a clinical trial investigating the use of EES to enable motor functions after SCI. The 16-contact electrode array was initially positioned under fluoroscopic guidance. Then, EES-evoked motor responses were recorded from select leg muscles and displayed in real time to determine electrode array proximity to spinal cord regions associated with motor activity of the lower extremities. Acceptable array positioning was determined based on achievement of selective proximal or distal leg muscle activity, as well as bilateral muscle activation. Motor response latencies were not significantly different between intraoperative recordings and post-operative recordings, indicating that array positioning remained stable. Additionally, EES enabled intentional control of step-like activity in both subjects within the first 5 days of testing. These results suggest that the use of EES-evoked motor responses may guide intraoperative positioning of epidural electrodes to target spinal cord circuitry to enable motor functions after SCI.

## Introduction

Severe spinal trauma may result in spinal cord injury (SCI) with permanent, devastating loss of motor, sensory, and autonomic functions.^1,2^ Scientific evidence from animal models of SCI and from early-phase clinical studies have reported positive outcomes such as decreased initial injury severity by pharmacological interventions,^3^ repair of damaged spinal cord tissues by implantation of biomaterials and engineered cells at the injury site,^4–6^ or direct signal transmission through or around the injury using state-of-the-art technology to interface with other functional neural pathways.^7–12^ Despite these promising results, translational efforts have yet to achieve significant functional gains across the population of humans with SCIs.^13^

Initial reports using epidural electrical stimulation (EES) applied to the dorsal surface of the lumbosacral spinal cord demonstrated that EES could induce tonic and rhythmic leg muscle activity both in animal models of complete SCI^14–18^ and in humans with chronic paraplegia attributed to severe SCI.^19–21^ The capacity of EES to facilitate intentional movement in an individual with motor complete SCI was first reported by Harkema and colleagues in 2011^22^ and subsequently replicated by the same investigators in 3 additional subjects.^11^ In total, 4 males diagnosed with motor complete paraplegia at least 2 years before study enrollment were implanted with an EES system over the lumbosacral spinal cord. After several months of locomotor training with EES, all 4 subjects could intentionally control leg movements while supine with EES on. Additionally, EES enabled subjects to stand for prolonged periods of time. While attempting to replicate the results reported by Harkema and colleagues,^11,22^ we reported that EES enabled intentional control of lower extremity movements and standing within the first 2 weeks of EES in a subject diagnosed with motor complete paraplegia,^12^ and over the course of 43 weeks of multi-modal rehabilitation, he achieved independent stepping in the presence of EES.^23^

Detailed descriptions of the motor functions that were enabled by EES have been reported by studies of humans with chronic, motor complete,^11,12,22^ and incomplete^24^ paraplegia, as well as motor complete tetraplegia.^25^ Despite small sample sizes, these positive outcomes, combined with investigations of EES to reporting improved cardiovascular,^26,27^ respiratory,^28–31^ and autonomic^32^ function as well as improved body composition^33^ after SCI, demonstrate that EES holds considerable potential as a therapeutic intervention after SCI. To date, the scientific literature has described EES-enabled functions and the stimulation patterns, electrode configurations applied, and motor training regimens used enable functions.^22,34–36^

Previous investigations using EES in humans with SCI described the use of intraoperative X-ray guidance to identify vertebral levels T11–L1 as the target location for insertion of an electrode array.^22^ However, X-ray imaging does not provide information regarding electrode proximity to spinal cord circuitry that enables function.^37^ Previous reports have briefly described the use of intraoperative electrophysiological monitoring to guide electrode array implantation over the lumbosacral spinal cord.^12,22^ However, the surgical approach to position the epidural electrode array, intraoperative electrophysiological techniques used to guide electrode positioning, and electrophysiological outcomes generated have yet to be described in detail. Herein, we provide a detailed description of the surgical approach with results from intraoperative electrophysiology of EES-evoked motor responses from leg muscles indicating appropriate positioning of the EES electrode array to enable motor functions, such as step-like lower extremity movement, after recovery from EES implantation.

## Methods

This study was performed under the approval of the Mayo Clinic Institutional Review Board with a U.S. Food and Drug Administration Investigational Device Exemption (IDE G150167), and all experiments were performed in accord with the regulations and guidelines of these regulatory bodies.

### Subject descriptions

Two males diagnosed with a sensorimotor complete, American Spinal Injury Association Impairment Scale Grade A (AIS-A) SCI^38^ were enrolled in this clinical trial (NCT02592668), and both provided written, informed consent to experimental procedures. Subject 1 was a 26-year-old male who sustained a T6 SCI during a snowmobile accident 3 years before study enrollment. Subject 2 was a 37-year-old male who sustained a T3 SCI from a fall 6 years before study enrollment. Data from subject 1 have been previously reported^12^; however, all data and analysis demonstrated in this report were not previously published.

### Motor training before to epidural electrical stimulation system implantation

In order to confirm that rehabilitation alone would not result in functional recovery, both subjects performed motor training for 6 months before implantation of the EES system. A detailed description of pre-surgical motor training for subject 1 was previously reported.^12^ Briefly, subjects underwent approximately 60 motor training sessions performed over 22 weeks for approximately three sessions per week by a team of physical therapists and kinesiologists. Motor training sessions consisted of approximately 15 min of lower extremity stretching, 45 min of locomotor training on a treadmill, and 30 min of balance and task-specific strengthening exercises. All motor training activities were performed with body weight support and trainer assistance provided as needed.

### Surgical approach to the spinal canal

After general anesthesia, subjects were positioned prone using a Jackson surgical table and Mayfield head clamp. General anesthesia was maintained by intravenous delivery of propofol and fentanyl. Inhalation agents and neuromuscular blocking agents were avoided for purposes of intraoperative electrophysiological monitoring of spinal sensorimotor activity during EES trialing.

A midline incision that spanned vertebrae T11–L2 was made based on palpation of anatomical landmarks and fluoroscopy. After initial exposure, surgical dissection of back musculature continued through the lumbodorsal fascia to the level of the spinous processes in an avascular plane using electrocautery. Once the spinous processes were exposed, vertebral level was confirmed intraoperatively by inserting a radio-opaque marker in proximity to the spinous process followed by fluoroscopy. Next, subperiosteal dissection of the muscle from the spinous process down to the lamina and then laterally toward the facets was performed. Then, the T11–12 and L1–2 interspinous ligaments were severed and the spinous processes of T12 and L1 were removed with a rongeur. The facet joints of both locations were preserved to ensure spinal stability. The laminae were then thinned with a 4-mm diamond drill bit and removed by rongeurs. Finally, the ligamentum flavum was dissected free from the underlying dura.

### Initial positioning of the epidural electrical stimulation electrode array

**FIG. 1.**
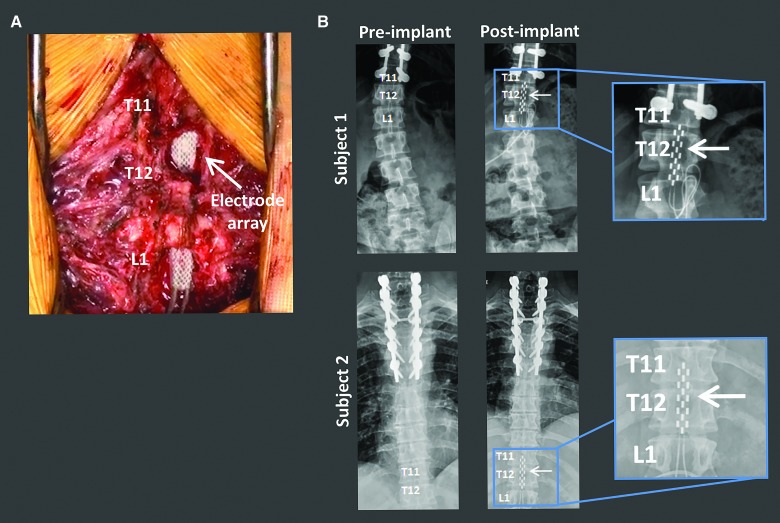
Surgical implantation of the EES electrode array spanning the lumbosacral spinal cord. (**A**) Intraoperative image of the location of the EES array at the T11–L1 vertebral levels. (**B**) Anterior-posterior X-ray of each subject before and after EES electrode array implantation. Subject 1 imaging was captured in a seated position. Subject 2 imaging was captured while lying supine. Inserts depict zoomed in view of the EES array. EES, epidural electrical stimulation.

A 16-contact epidural electrode array (Specify 5-6-5; Medtronic, Fridley, MN) was passed through the T12–L1 laminectomy and directed rostrally along the dorsal midline of the dura to span the T11–L1 vertebrae (Fig. 1). Insertion of the electrode to the adequate level was visualized through the L1–2 opening. T12–L1 exposure provided access to the caudal portion of the array while the L1–2 opening provided access to the rostral portion, allowing array visualization and mediolateral array manipulation. To determine array integrity and compliance with surrounding tissue, the array was connected to an external pulse generator to capture impedance values at each electrode contact. Upon initial implantation, intraoperative fluoroscopy was used to confirm the electrode array spanned the appropriate vertebral levels.

### Intraoperative electrophysiology

After induction but before surgical exposure, 13-mm subdermal electrodes were placed over selected muscles of the lower limbs in a muscle to tendon recording montage. The active electrode was placed over the midpoint of the appropriate muscle with the reference electrode over the distal tendon. A ground electrode was placed in the anterior thigh.

Recording electrodes were placed bilaterally into the rectus femoris (RF), vastus lateralis (VL), medial hamstring (MH), tibialis anterior (TA), medial gastrocnemius (MG), and soleus (SOL). These muscles were selected to assess selectivity of EES to activate proximal or distal muscle activation as well as to assess symmetry of activation between the legs.^34^ Once the array was inserted, minor changes in position were guided based on leg muscle intramuscular electromyography (EMG) recordings of EES-evoked motor responses. EES-evoked motor response recordings were captured during multiple EES configurations and stimulation parameters (0–10 V, 0.5–1.0 Hz), progressing from rostral to caudal and wide field to local. Additionally, EMG recordings were captured from the paraspinal muscles to record a stimulation artifact, which acted as the sweep trigger for real-time display of EES-evoked motor responses.

### Lead and battery placement

After confirmation of electrode position by electrophysiology, the electrode array was secured to the fascia and leads were tunneled subcutaneously to the right upper abdomen where a small subcutaneous pocket was made to house the lead wires. After meticulous hemostasis and copious irrigation with a bacitracin solution, the posterior wounds were closed in anatomical layers. Subjects were then positioned on the operating table in a lateral decubitus position. After prepping and draping the side and lateral abdomen, the lead wires were externalized and a pocket was made to insert the implantable pulse generator (RestoreUltra SureScan MRI Neurostimulator, Model 97712; Medtronic) in a pre-determined area that would least interfere with daily activities. The neurostimulator was connected to the lead wires and inserted into the subcutaneous pocket. The anterior wound was then irrigated with bacitracin solution and closed in anatomic layers.

### Post-operative electrophysiology

After 3 weeks of rest, subjects returned to the laboratory to record EES-evoked motor responses from leg muscles while lying supine using EES parameters that were applied during intraoperative recordings (0–10 V, 0.5–1.0 Hz).

### Epidural electrical stimulation–enabled motor activity

During the first 5 days of testing with EES, both subjects attempted to perform motor tasks in the presence of EES. Tasks included attempts to stand, control flexion/extension lower extremity movements while supine or side-lying, and move their legs in response to an audio or visual cue. All tasks were performed with trainer assistance provided as needed. These tasks were performed with the primary goal of identifying EES configurations and stimulation parameters that enabled intentional control of lower extremity motor activity.^12^

To determine whether EES could enable intentional control of coordinated, robust leg muscle activity, subjects were positioned side lying with the top leg suspended using non-elastic nylon fabric support slings to allow gravity-neutral movement to identify EES parameters that enabled intentional control over robust rhythmic muscle activity to generate step-like movements.^39,40^ A range of electrode configurations and voltage intensities were tested based on past literature in combination with intra- and post-operative interpretations of EES-evoked motor responses. EES voltage intensity was increased until volitional control over leg muscle activity and movement was observed.^11,20,22,41^ Skin-surface EMG recordings were captured over the same leg muscles as intra- and post-operative EES-evoked motor response recordings. For subject 2, goniometer recordings of knee joint changes were synchronized to EMG recordings.

### Statistical analysis

EMG and goniometry data were collected at a sampling rate of 4 kHz (PowerLab; ADInstruments, Austin, TX) and analyzed using custom code written in MATLAB (Version R2015a; The MathWorks, Inc., Natick, MA). Notch (60 Hz) and bandpass (20–1000 Hz) filters were applied to EMG recordings to reduce environmental artifacts. EES-evoked motor response latencies were calculated as the time from stimulus pulse to the onset of the motor response of each muscle; defined as the time at which the response amplitude exceeded ±3 standard deviations (SDs) from baseline EMG values during quiescent recordings. Mean and SD values were calculated from five consecutive stimuli. Root mean square (RMS) envelopes were calculated from full-wave rectified EMG using a moving window of 200 samples and an overlap of 50 samples. Area under the curve values were calculated by taking the trapezoidal integral of the calculated RMS EMG data. The Shapiro-Wilk method was used to test for normal distributation of data. For normally distributed data, statistically significant differences between motor response amplitudes were calculated using standard parametric *t*-tests to compare muscle activity. Data that were not normally distributed were analyzed using the Wilcoxon signed-rank test. To determine statistically significant differences in motor activity across muscles, a one-way analysis of variance (ANOVA) was followed by a multiple comparisons test.

## Results

### Electrode array positioning guided by intraoperative epidural electrical stimulation–evoked motor responses

**FIG. 2.**
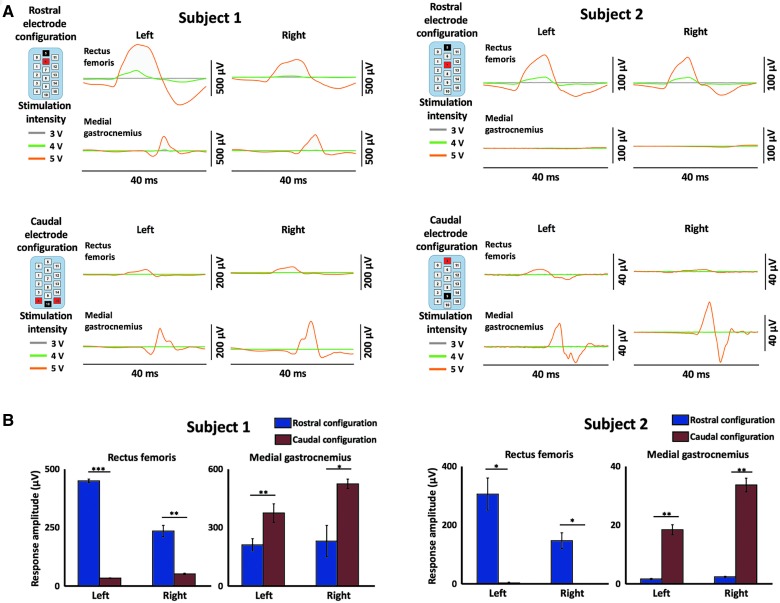
Intraoperative EES-evoked motor response recordings demonstrates selective activation of rostral and caudal spinal circuitry. (**A**) EES-evoked responses during rostral electrode array configurations demonstrate proximal muscle activation (rectus femoris) and during caudal electrode array configurations demonstrate distal muscle activation (medial gastrocnemius). Each line represents the average evoked response to stimulation over five stimulations. Gray, green, and orange lines represent stimulation at 3, 4, and 5 volts, respectively. Stimulation occurs at the 0 time point of each plot. Stimulation configuration is shown in the upper left of each figure; black = cathode, red = anode. (**B**) Bar plots displaying maximum evoked response in given muscles from (A) with amplitude calculated as maximum – minimum response. Blue bars indicate rostral configurations and red bars indicate caudal configurations. * = <0.05; ** = <0.01; *** = <0.001. EES, epidural electrical stimulation; NS, not significant; R, right; L, left.

Initial positioning of the electrode array within the T11–L1 vertebral region was performed using anatomical landmarks and fluoroscopy (Figure 1). Activation of select electrodes on the array evoked distinct motor responses. Specifically, positioning the active electrodes at the rostral region of the array resulted in significantly higher EES-evoked motor response amplitudes in proximal leg muscles compared to EES-evoked motor response amplitudes generated when active electrodes were selected at the caudal region of the array (Fig. 2; subject 1, *p* = 0.0002 and *p* = 0.0042 for left and right leg, respectively; subject 2, *p* = 0.0101 and *p* = 0.0110 for left and right leg, respectively). Stimulation through caudal electrode configurations resulted in distal muscle EES-evoked motor response amplitudes that were significantly higher than rostral positioning of active electrodes (Fig. 2; subject 1, *p* = 0.0055 and *p* = 0.0222 for left and right leg, respectively; subject 2, *p* = 0.0038 and *p* = 0.0017 for left and right leg, respectively).

**FIG. 3.**
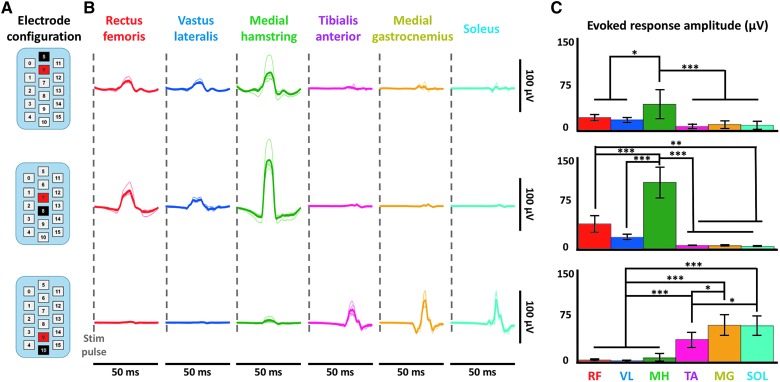
EES-evoked motor responses activate specific muscle circuitry intraoperatively. (**A**) EES-evoked responses during stimulation of the rostral, intermediate, and caudal portions of the EES array are demonstrated in six muscles (rectus femoris, vastus lateralis, medial hamstring, tibialis anterior, medial gastrocnemius, and soleus) from subject 1. Stimulation occurs at the start of each EES-evoked response trace as indicated by the gray dashed line. Dark traces are average of five individual responses that are shown in light traces. Data shown are from motor threshold responses. EES electrode array configuration is shown in the upper left; black = cathode, red = anode. (**B**) Bar plots displaying maximum EES-evoked responses in given muscles (RF = rectus femoris, VL = vastus lateralis, MH = medial hamstring, TA = tibialis anterior, MG = medial gastrocnemius, and SOL = soleus) from (A) with amplitude calculated as maximum – minimum EES-evoked response. Statistical significance was calculated by a one-way ANOVA followed by a multiple comparisons test and is shown above the bar plots. * = <0.05; ** = <0.01; *** = <0.001; no stars indicates not significant. ANOVA, analysis of variance; EES, epidural electrical stimulation.

Stimulating midline electrodes on the array at the rostral, middle, and caudal regions resulted in voltage-dependent selective activation of all six recorded muscles (Fig. 3A). Stimulation at the rostral portion of the array resulted in significantly higher amplitude MH activity compared to all five other muscles (Fig. 3B,C). Stimulation of the middle portion of the array resulted in significantly higher response amplitudes in the RF and MH (Fig. 3B,C). Stimulation at the caudal portion of the array resulted in significantly higher responses in the distal muscles compared to the proximal muscles, with significantly higher responses in the MG and SOL compared to the TA (Fig. 3B,C).

**FIG. 4.**
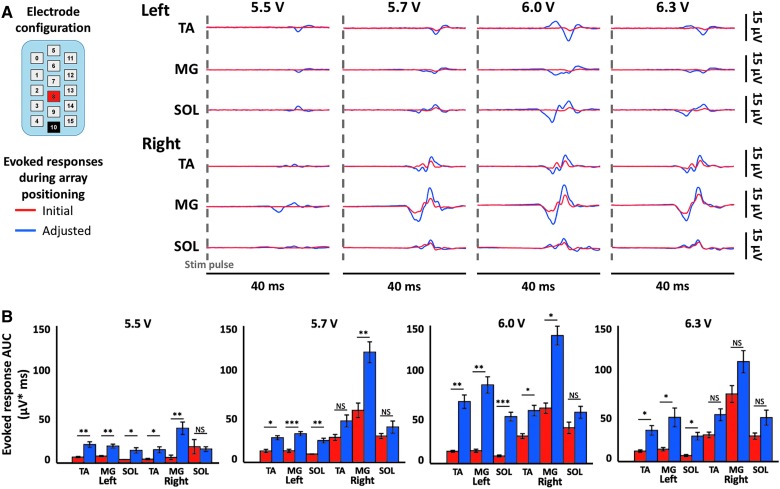
Electrode location adjustment guided by intraoperative EES-evoked motor response recordings. (**A**) Intraoperative data from subject 2 using a caudal, symmetric (−10/+8) configuration as displayed. Bilateral electromyography (EMG) data from three bilateral distal muscles are shown (TA = tibialis anterior, MG = medial gastrocnemius, and SOL = soleus). Each line is an average of five motor-evoked potentials where stimulation occurs at the start of each trace. Data are shown while increasing the stimulation intensity incrementally from 5.5 to 6.3 V before and after shifting of the array during surgery. (**B**) Area under the curve of the EES-evoked responses at the four different voltages. * = <0.05; ** = <0.01; *** = <0.001; NS = not significant. Red indicates data before array shift. Blue indicates data after shift. EES, epidural electrical stimulation.

To determine whether the array was positioned to allow symmetrical activation of bilateral leg muscles, electrodes that were midline on the array were activated. In subject 2, initial positioning of the electrode array resulted in EES-evoked motor responses that were asymmetric in response amplitude. Asymmetry was characterized by increasing motor response amplitudes in right leg muscle recordings as EES voltages increased. However, unremarkable changes in motor response amplitudes were observed in left leg muscle recordings as EES voltages were incrementally increased (Fig. 4A). In response to this observed asymmetry, the electrode array was shifted to the left by 2 mm. Lateral adjustment in array positioning resulted in both the left and right leg muscles becoming active at lower EES voltages with significantly greater evoked motor response amplitudes at multiple stimulation voltages in all left and right leg muscles, with the exception of the right SOL (Fig. 4B).

### Characteristics of epidural electrical stimulation–evoked motor responses intraoperatively and at 3 weeks post-surgery

**FIG. 5.**
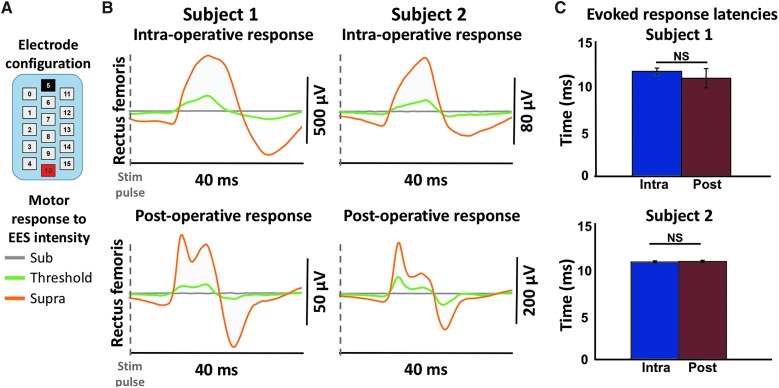
Comparison of intraoperative and post-operative EES-evoked motor responses. (**A**) EES electrode array configuration used intraoperatively and post-operatively; black = cathode, red = anode. (**B**) EMG (electromyography) data are shown from the left rectus femoris of both subjects recorded intraoperatively and post-operatively using the same electrode configuration for each subject after 3 weeks of recovery from surgery. Each trace represents the average of five consecutive evoked motor responses at each EES voltage intensity. Data are shown at subthreshold levels of stimulation when no response was observed, at motor threshold where the first appearance of motor activity was observed, and at the maximum level of stimulation. These voltage values ranged from 3 to 6 V. (**C**) Latency of the suprathreshold evoked response in the left rectus femoris in both subjects both intraoperatively and at 3 weeks post-operatively. No significant difference was found between intraoperative and post-operative latency for either subject. EES, epidural electrical stimulation; NS, not significant.

Three weeks after EES implantation, subjects were positioned supine and instructed to relax while EES-evoked motor responses were recorded using intraoperative electrode configurations. Leg muscle activity was recorded using EES voltages that were subthreshold, threshold, and suprathreshold with respect to evoked motor responses (Fig. 5A). EES-evoked motor responses differed in deflection shape, peak amplitude, and number of peaks between intra- and post-operative recording time points. In the RF, one peak in motor response amplitude was observed intraoperatively, and two distinct peaks were observed post-operatively in both subjects (Fig. 5B). However, latencies from the stimulus pulse to motor response onset in the RF (subject 1, mean = 11.60 ± 0.38 and 10.85 ± 1.07 ms for intra- and post-operative, respectively; subject 2, mean = 11.10 ± 0.14 and 11.15 ± 0.14 ms for intra- and post-operative, respectively) were statistically insignificant between intra- and post-surgical time points across time (subject 1, p = 0.30; subject 2, *p* = 0.62; Fig. 5C).

### Intentional control of epidural electrical stimulation–enabled step-like activity while side-lying

**FIG. 6.**
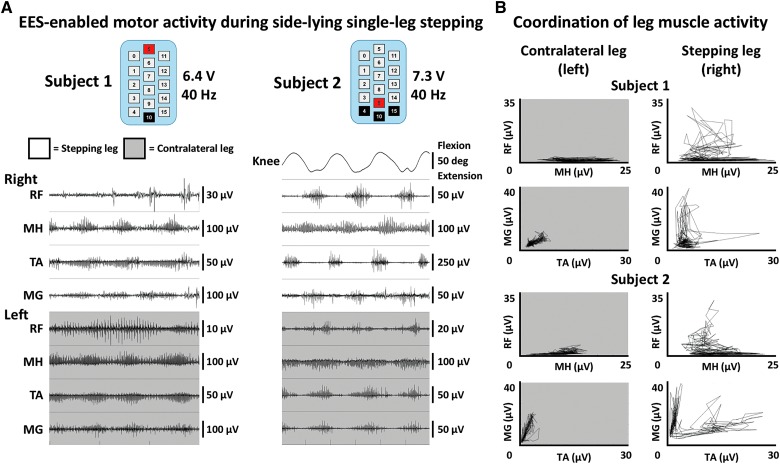
Intentional control of rhythmic movement while side-lying. (**A**) EMG (electromyography) and goniometer data from both subjects are shown while each subject intentionally attempted to generate EES-enabled step-like movements of their right leg. EMG data were recorded bilaterally from rectus femoris (RF), medial hamstring (MH), tibialis anterior (TA), and medial gastrocnemius (MG). Subject 2 wore sagittal knee goniometers during testing to quantify leg motion. Stimulation configurations were chosen that allowed for optimal movement. Pulse width and frequency were held constant at 210 μs and 40 Hz, respectively. Voltage was incrementally increased until subjects displayed ability to intentionally control leg movements. White background indicates the leg that was supported in a gravity-neutral position in order to move freely. Gray background indicates the leg that was resting on a table and limited with respect to movement capability. (**B**) Muscle coordination plots from the same data as part (A). Root mean square (RMS) envelopes of the EMG data were calculated and antagonistic muscles are plotted against one another to demonstrate patterns of coordination. Note that a more normal L-shaped (reciprocal) coordination patterned was generated when the leg was suspended and freed of surface tension. EES, epidural electrical stimulation.

When positioned side-lying with one leg suspended in a gravity-neutral position by a sling system, both subjects demonstrated the ability to intentionally initiate, maintain, and terminate EES-enabled robust, rhythmic leg muscle activity, and step-like leg movements. First, motor threshold was detected as the voltage that resulted in EMG activity, which was achieved at 3.5 V for subject 1 and 2.5 V for subject 2. In both subjects, when voltage increased, rhythmic step-like activity was enabled. As shown in Figure 6, both subjects were able to make five consecutive steps while attempting step-like movements of the right leg. These consecutive steps were characterized by reciprocal, EMG activity in all muscle recordings of the right leg which was free to move in a gravity-neutral plane by the support sling (Fig. 6B). For subject 2, right knee goniometer recordings were synchronized to EMG, and demonstrated movement patterns that coincided with bursting EMG activity in the leg muscles. Supplementary Video 1 (see online supplementary material at http://www.liebertpub.com) shows the range of movement and muscle activity observed during these attempts. These data and video are from the fifth and second day of EES testing for Subjects 1 and 2, respectively. Without EES, subjects were unable to intentionally generate any sustained, coordinated muscle activity in their legs.

## Discussion

EES-enabled return of intentional leg movement, standing, and independent stepping has been described in individuals with SCI who have performed motor training in the presence of EES.^11,12,23,35,36,42,43^ These scientific observations have created a large interest within the SCI community and a sense of urgency in translating EES into an effective rehabilitation tool for individuals with SCI-related deficits. As the scientific understanding of EES-enabled function continues to improve, along with the development of next-generation neurotechnology to interface with the spinal cord, the therapeutic potential of EES continues to increase. Here, we have detailed the surgical procedures of EES implantation for use as a foundation for future clinical investigations seeking to implant EES systems after SCI. We also provided the approach of using intraoperative EES-evoked motor responses to guide electrode array positioning and compared those motor response characteristics to post-surgical EES-evoked motor responses.

Intraoperative electrophysiological monitoring is a valuable technique for real-time assessment of nervous system health during surgery.^44^ Intraoperative monitoring in subjects with SCI has been primarily used as a technique to identify impending or ongoing injury and to decrease the risk of spinal cord insult during surgical procedures. A systematic review has found that the procedure is sensitive and specific for detecting intraoperative injury, and a separate Monte Carlo simulation study found a 49% reduction in risk in post-operative complications.^45,46^ Here, we demonstrated the utility of intraoperative monitoring to guide EES electrode positioning for post-surgical EES-enabled motor activity in 2 subjects undergoing EES procedures.

Animal models have shown that localization of epidural electrodes relative to the level of the dorsal roots plays a critical role in determining the intensity of stimulation required to activate select spinal circuitry, and this dependency can vary at a submillimeter resolution.^37^ Monitoring intraoperative electrophysiological responses in real time allowed for precise placement of the epidural electrode array. By applying stimuli at multiple different regions of the spinal cord using specific electrode configurations, selective activation of spinal sensorimotor networks was achieved and interpreted from EES-evoked motor response recordings. Rostral configurations preferentially activated proximal muscles, and caudal configurations activated distal muscles in both subjects (see Fig. 2). Targeting EES to selectively activate these motor pools proved useful to confirm that the electrode array spanned the intended region of the lumbosacral spinal cord in subject 1. Further, when stimulation was then applied midline at rostral, intermediate, and caudal portions of the array, specific spinal segments were likely activated and resulted in higher amplitude EES-evoked responses (see Fig. 3). Previous reports indicate that stimulation of these regions activates L1–S1 spinal segments.^12,34^ Stimulation at the rostral portions of the cord resulted in statistically larger responses in the MH compared to all other muscles. Stimulation at the intermediate portions of the array resulted in increased response amplitude in the MH and RF muscles compared to the distal muscles. Finally, stimulation at the caudal portion resulted in increased response amplitude in the distal muscles (TA, MG, and SOL). Based on the array location, the rostral, intermediate, and caudal stimulations were likely over the L1/L2, L3/L4, and L5/S1 spinal segments, respectively. These results suggest that spatially restricted stimulation ensures that localization of active electrodes on the array can activate the most proximal or distal muscles of the leg, or excite select intermediate muscles.

In conjunction with the use of spatially restricted stimulation to target specific leg motor activity, the symmetry of the responses was also investigated. In subject 2, the electrode array was initially found to be off-set to the right as determined by a symmetric electrode configuration, which generated activity in only the right leg muscles (see Fig. 4). This asymmetry was detected and quantified using intraoperative EES-evoked motor response recording equipment, and required a lateral shift of 2 mm by the neurosurgeon in order to increase symmetry of evoked motor response amplitudes across left and right leg muscles.

In addition to providing real-time monitoring of the EES-evoked motor responses during surgery, intraoperative monitoring predicts post-operative evoked motor response characteristics and establishes the groundwork for optimizing EES stimulation parameters and active electrode configurations to be used during post-surgical EES-enabled motor activities (see Fig. 5). EES-evoked motor responses varied in amplitude and shape, possibly attributed to a range of factors including depth of anesthesia, intramuscular versus skin electrode, and body positioning. However, evoked motor response latencies were statistically similar across intra- and post-operative environments, indicating that intraoperative recordings of EES-evoked motor responses can be used to establish characteristics of sensorimotor networks activated by EES.

The evoked potentials observed during surgical procedures and on the first day of stimulation after surgery provide considerable clarity of the functional connectivity from the dorsum of the spinal cord to ventral motor pools and downstream neuromuscular circuitry. These electrically evoked motor responses are a critical component used to assess sensorimotor spinal network activity after SCI, and they demonstrate the potential of EES to achieve effective recruitment patterns of muscle activity. Further, we demonstrate that within 5 days of testing, it is possible to achieve coordinated and robust motor output to generate leg muscle activity that can be intentionally modulated to create step-like movements (see Fig. 6). This demonstrates a fundamentally important result in the rehabilitative strategy for paralyzed individuals. Specifically, the unweighted condition reflects the ability of a novel supraspinal-spinal connectome to generate an impressive degree of agonist-antagonist coordination at such an early stage after spinal networks are neuromodulated. Theoretically, then, the challenge during rehabilitation is to progressively improve the ability of the muscular component to generate the desired torques in the presence of a coordinated pattern of activation to achieve a given movement, and that this may be achieved through task-specific training.

Although the results we presented from these 2 subjects suggest that intra-operative EES-evoked motor responses foreshadow EES-enabled motor activity performance post-surgery, they do not directly demonstrate how these single evoked potentials can serve as a biomarker of the most effective stimulation parameters to perform step-like behavior. There are multiple factors that contribute to the complexity of interactions between EES and the dynamic state of sensorimotor spinal network excitability. This complex state of excitability prohibits a direct correlation of EES-evoked motor potential characteristics to EES-enabled motor functions. For example, changing the stimulation from a single pulse to a train of pulses or adjustment of stimulus frequency cannot readily define the optimal stimulation parameters for generating a desired motor function. Another factor that makes the translation from evoked potentials to motor outcomes difficult is that the responsiveness of the spinal network intraoperatively and soon after implantation is likely to change substantially as motor function improves. One of the most obvious differences when stimulating single evoked responses versus continuous stimulation is that the spinal networks activated in these two conditions are dramatically different, quantitatively and qualitatively. For example, the behavioral responses reflect the engagement of proprioception and a return of supraspinal connectivity. With our currently limited understanding of the mechanisms underlying the functional connections that generate a motor behavior, it cannot be expected that the response of the final common pathways to each motor pool would be similar to a single evoked response compared to the ensembles of proprioceptive and supraspinal inputs projecting to spinal sensorimotor networks.

EES has shown promise to improve function previously lost attributed to SCI, and this study provides further evidence of the ability of EES to enable motor function in 2 subjects with motor complete SCI. Here, we demonstrate that intraoperative EMG recordings can be used to achieve adequate array positioning by spatially restricted stimulation, that similar spinal structures are stimulated intraoperatively, and post-operatively, and that stimulation of these structures can enable coordinated motor function within the first few days of EES. The surgical procedures and electrophysiological results presented lay the groundwork for future investigations using EES in humans with SCI.

## Supplementary Material

Supplemental data
